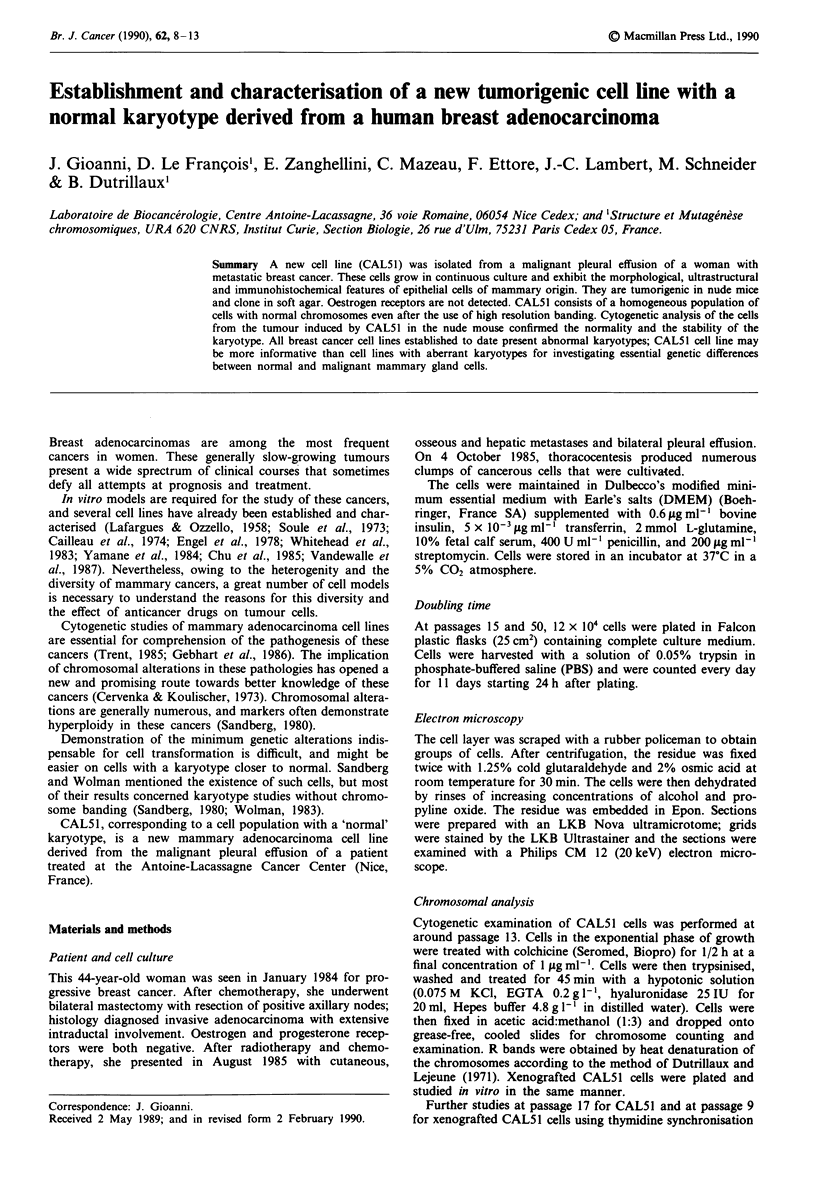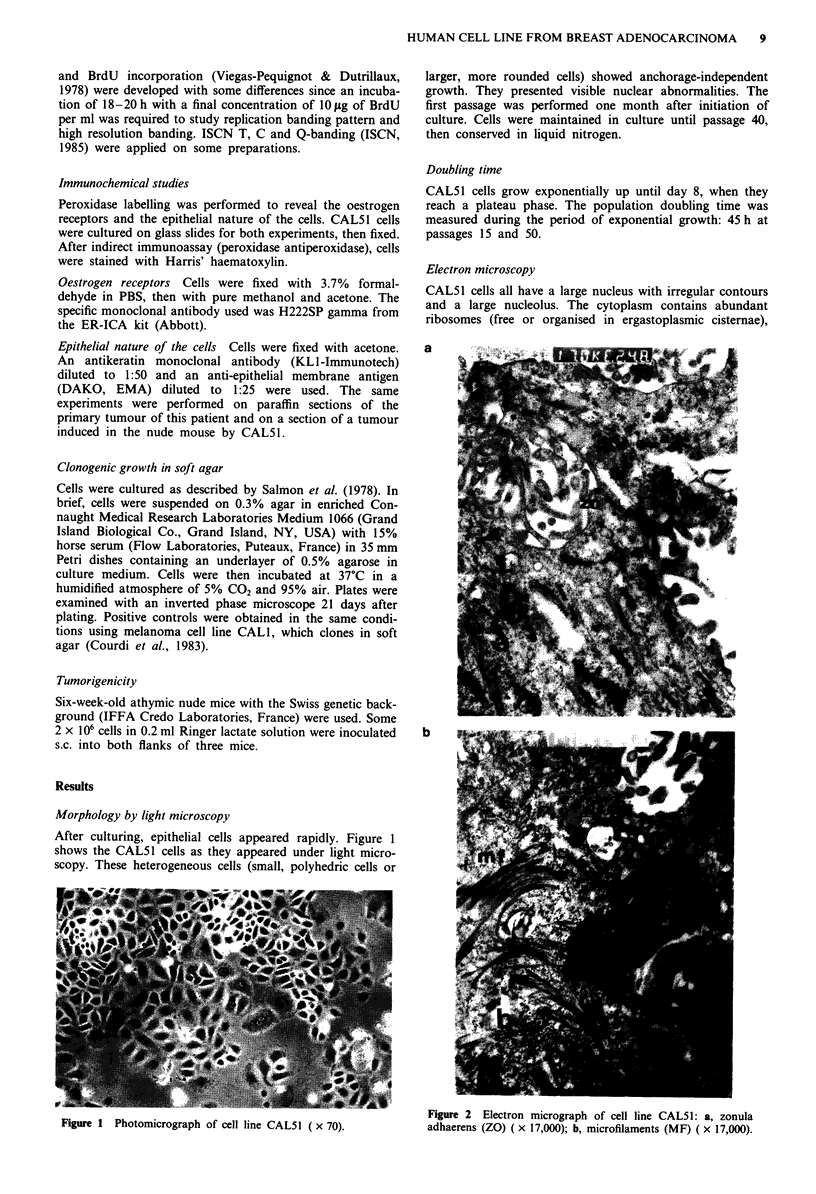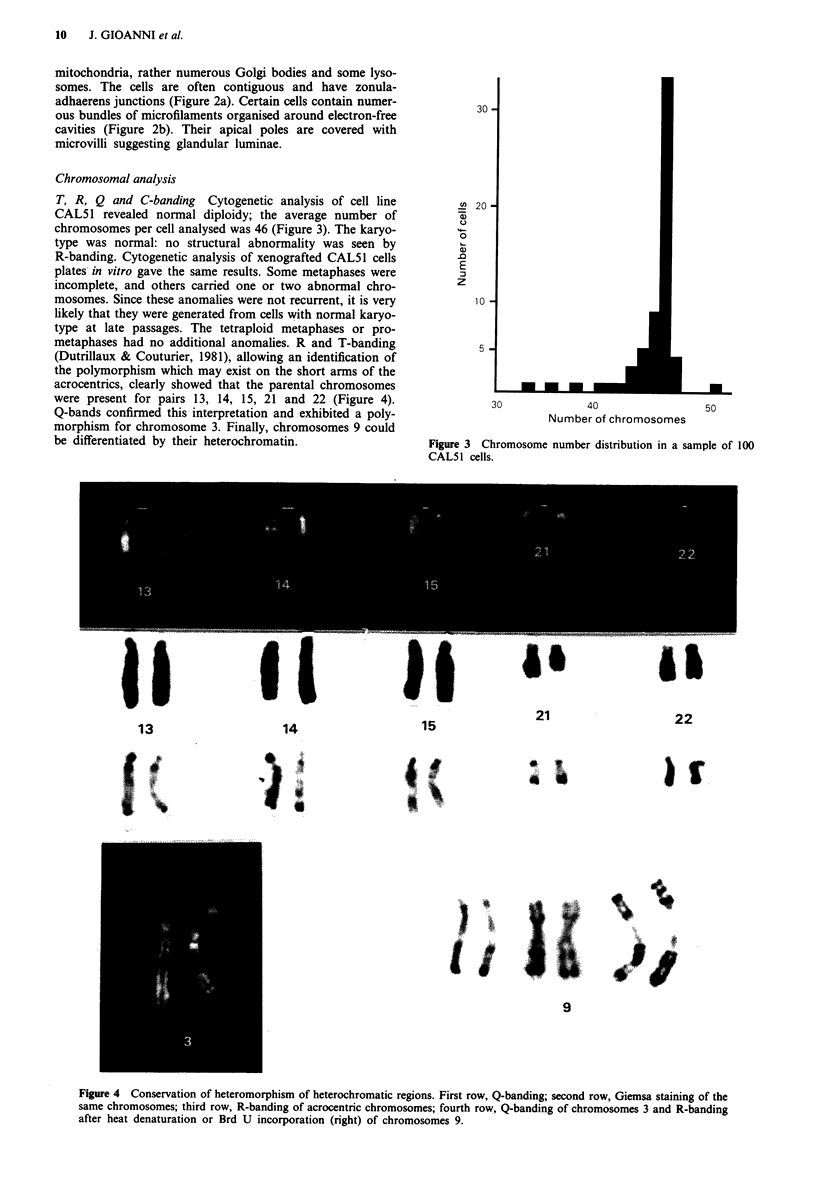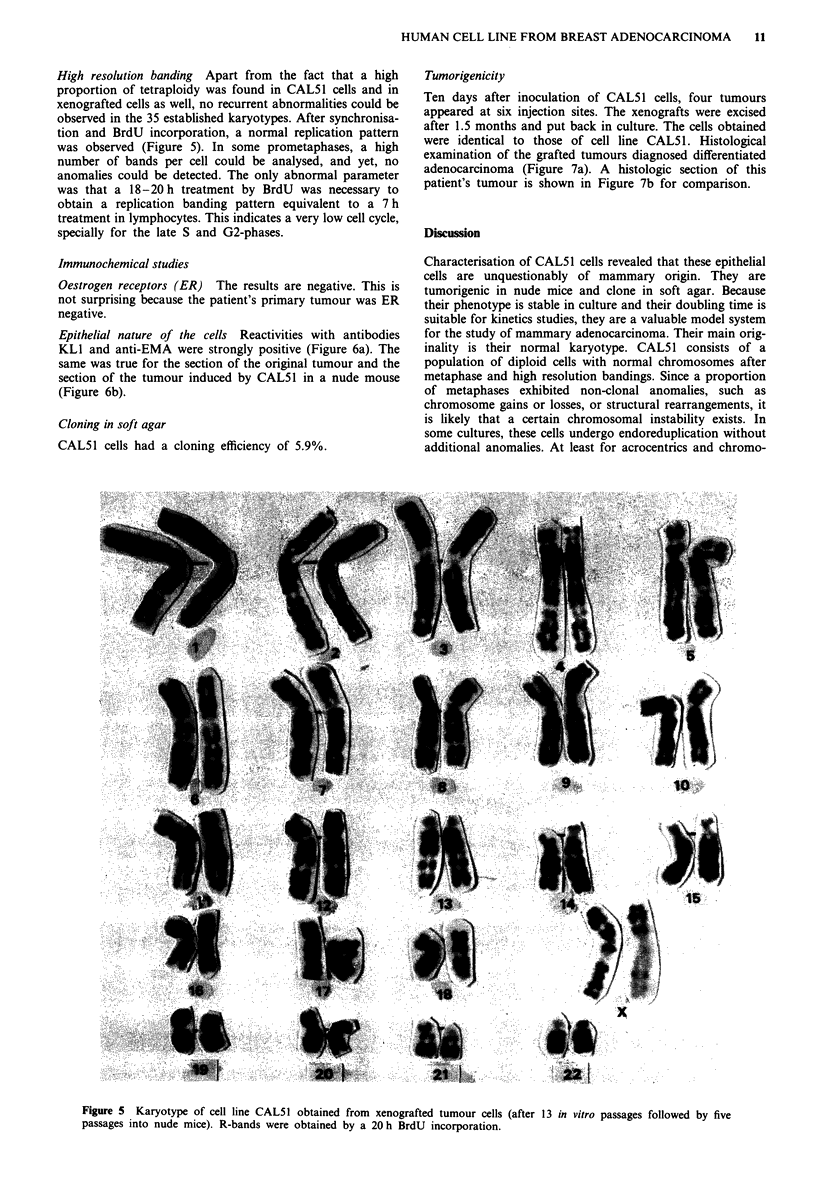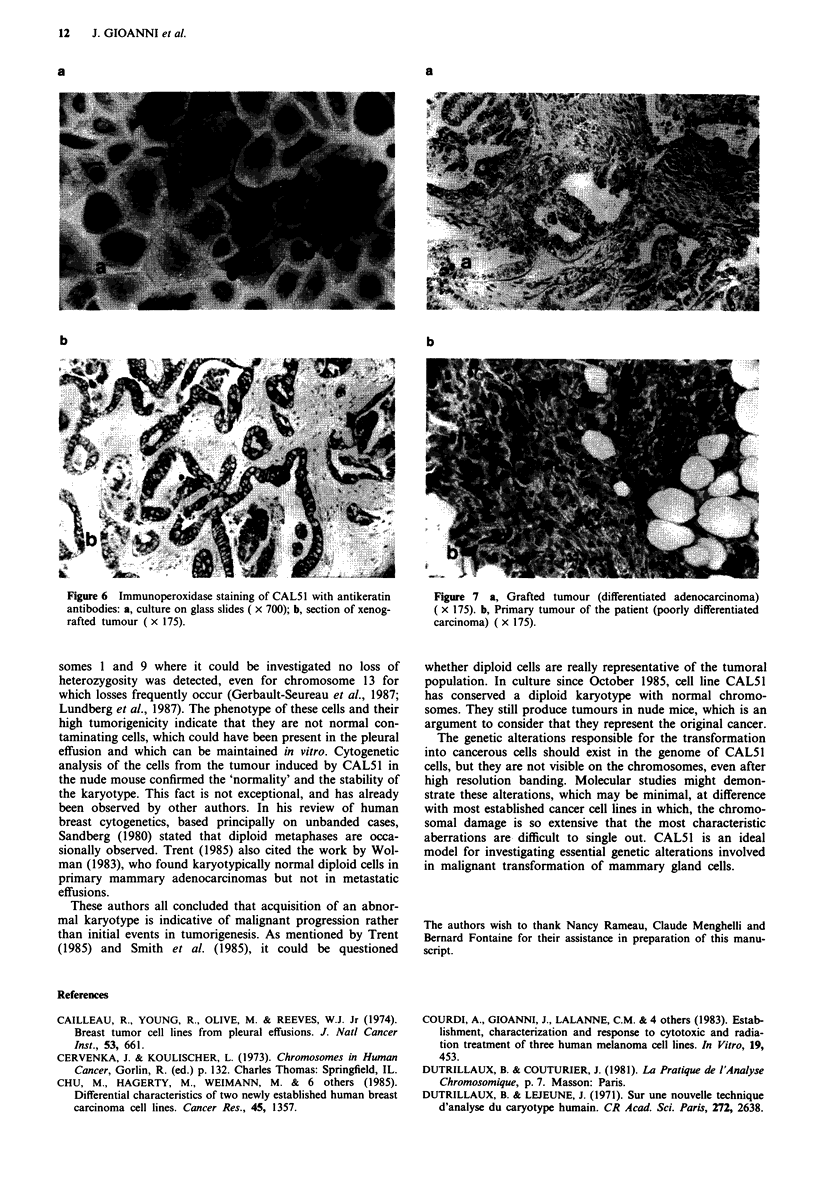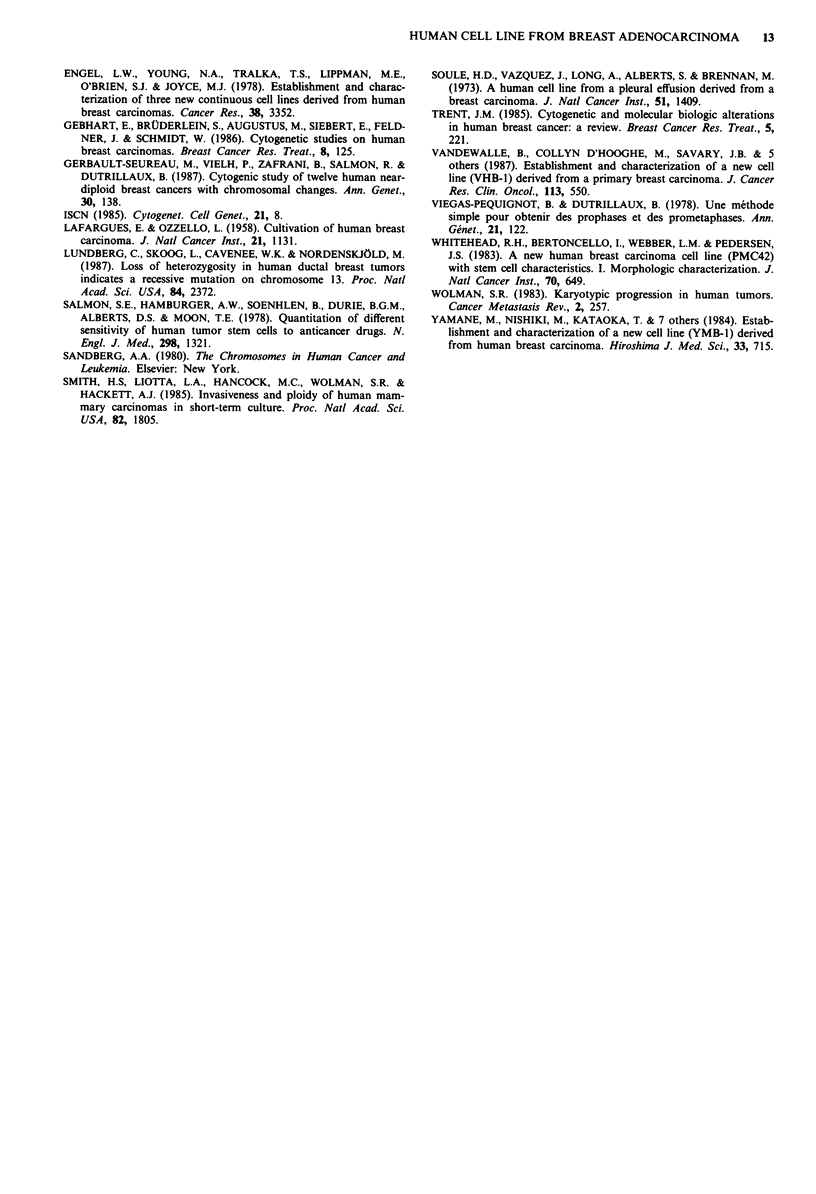# Establishment and characterisation of a new tumorigenic cell line with a normal karyotype derived from a human breast adenocarcinoma.

**DOI:** 10.1038/bjc.1990.219

**Published:** 1990-07

**Authors:** J. Gioanni, D. Le François, E. Zanghellini, C. Mazeau, F. Ettore, J. C. Lambert, M. Schneider, B. Dutrillaux

**Affiliations:** Laboratoire de Biocancérologie, Centre Antoine-Lacassagne, Nice, France.

## Abstract

**Images:**


Br. J. Cancer (1990), 62, 8-13 © Macmillan Press Ltd., 1990

### Establishment and characterisation of a new tumorigenic cell line with a normal karyotype derived from a human breast adenocarcinoma

J. Gioanni, D. Le François[1], E. Zanghellini, C. Mazeau, F. Ettore, J.-C. Lambert, M. Schneider & B. Dutrillaux[1]

*Laboratoire de Biocancérologie, Centre Antoine-Lacassagne, 36 voie Romaine, 06054 Nice Cedex; and [1]Structure et Mutagénèse chromosomiques, URA 620 CNRS, Institut Curie, Section Biologie, 26 rue d'Ulm, 75231 Paris Cedex 05, France.*

**Summary** A new cell line (CAL51) was isolated from a malignant pleural effusion of a woman with metastatic breast cancer. These cells grow in continuous culture and exhibit the morphological, ultrastructural and immunohistochemical features of epithelial cells of mammary origin. They are tumorigenic in nude mice and clone in soft agar. Oestrogen receptors are not detected. CAL51 consists of a homogeneous population of cells with normal chromosomes even after the use of high resolution banding. Cytogenetic analysis of the cells from the tumour induced by CAL51 in the nude mouse confirmed the normality and the stability of the karyotype. All breast cancer cell lines established to date present abnormal karyotypes; CAL51 cell line may be more informative than cell lines with aberrant karyotypes for investigating essential genetic differences between normal and malignant mammary gland cells.

Breast adenocarcinomas are among the most frequent cancers in women. These generally slow-growing tumours present a wide sprectrum of clinical courses that sometimes defy all attempts at prognosis and treatment.

*In vitro* models are required for the study of these cancers, and several cell lines have already been established and characterised (Lafargues & Ozzello, 1958; Soule *et al.*, 1973; Cailleau *et al.*, 1974; Engel *et al.*, 1978; Whitehead *et al.*, 1983; Yamane *et al.*, 1984; Chu *et al.*, 1985; Vandewalle *et al.*, 1987). Nevertheless, owing to the heterogenity and the diversity of mammary cancers, a great number of cell models is necessary to understand the reasons for this diversity and the effect of anticancer drugs on tumour cells.

Cytogenetic studies of mammary adenocarcinoma cell lines are essential for comprehension of the pathogenesis of these cancers (Trent, 1985; Gebhart *et al.*, 1986). The implication of chromosomal alterations in these pathologies has opened a new and promising route towards better knowledge of these cancers (Cervenka & Koulischer, 1973). Chromosomal alterations are generally numerous, and markers often demonstrate hyperploidy in these cancers (Sandberg, 1980).

Demonstration of the minimum genetic alterations indispensable for cell transformation is difficult, and might be easier on cells with a karyotype closer to normal. Sandberg and Wolman mentioned the existence of such cells, but most of their results concerned karyotype studies without chromosome banding (Sandberg, 1980; Wolman, 1983).

CAL51, corresponding to a cell population with a 'normal' karyotype, is a new mammary adenocarcinoma cell line derived from the malignant pleural effusion of a patient treated at the Antoine-Lacassagne Cancer Center (Nice, France).

### Materials and methods

### Patient and cell culture

This 44-year-old woman was seen in January 1984 for progressive breast cancer. After chemotherapy, she underwent bilateral mastectomy with resection of positive axillary nodes; histology diagnosed invasive adenocarcinoma with extensive intraductal involvement. Oestrogen and progesterone receptors were both negative. After radiotherapy and chemotherapy, she presented in August 1985 with cutaneous, osseous and hepatic metastases and bilateral pleural effusion. On 4 October 1985, thoracocentesis produced numerous clumps of cancerous cells that were cultivated.

The cells were maintained in Dulbecco's modified minimum essential medium with Earle's salts (DMEM) (Boehringer, France SA) supplemented with 0.6 $\mu g\ ml^{-1}$ bovine insulin, $5 \times 10^{-3} \mu g\ ml^{-1}$ transferrin, 2 mmol L-glutamine, 10% fetal calf serum, 400 U $ml^{-1}$ penicillin, and 200 $\mu g\ ml^{-1}$ streptomycin. Cells were stored in an incubator at 37°C in a 5% $CO_2$ atmosphere.

### Doubling time

At passages 15 and 50, $12 \times 10^4$ cells were plated in Falcon plastic flasks (25 $cm^2$) containing complete culture medium. Cells were harvested with a solution of 0.05% trypsin in phosphate-buffered saline (PBS) and were counted every day for 11 days starting 24 h after plating.

### Electron microscopy

The cell layer was scraped with a rubber policeman to obtain groups of cells. After centrifugation, the residue was fixed twice with 1.25% cold glutaraldehyde and 2% osmic acid at room temperature for 30 min. The cells were then dehydrated by rinses of increasing concentrations of alcohol and propyline oxide. The residue was embedded in Epon. Sections were prepared with an LKB Nova ultramicrotome; grids were stained by the LKB Ultrastainer and the sections were examined with a Philips CM 12 (20 keV) electron microscope.

### Chromosomal analysis

Cytogenetic examination of CAL51 cells was performed at around passage 13. Cells in the exponential phase of growth were treated with colchicine (Seromed, Biopro) for 1/2 h at a final concentration of 1 $\mu g\ ml^{-1}$. Cells were then trypsinised, washed and treated for 45 min with a hypotonic solution (0.075 M KCl, EGTA 0.2 $g\ l^{-1}$, hyaluronidase 25 IU for 20 ml, Hepes buffer 4.8 $g\ l^{-1}$ in distilled water). Cells were then fixed in acetic acid:methanol (1:3) and dropped onto grease-free, cooled slides for chromosome counting and examination. R bands were obtained by heat denaturation of the chromosomes according to the method of Dutrillaux and Lejeune (1971). Xenografted CAL51 cells were plated and studied *in vitro* in the same manner.

Further studies at passage 17 for CAL51 and at passage 9 for xenografted CAL51 cells using thymidine synchronisation

Correspondence: J. Gioanni.
Received 2 May 1989; and in revised form 2 February 1990.

and BrdU incorporation (Viegas-Pequignot & Dutrillaux, 1978) were developed with some differences since an incubation of 18–20 h with a final concentration of 10 µg of BrdU per ml was required to study replication banding pattern and high resolution banding. ISCN T, C and Q-banding (ISCN, 1985) were applied on some preparations.

### *Immunochemical studies*

Peroxidase labelling was performed to reveal the oestrogen receptors and the epithelial nature of the cells. CAL51 cells were cultured on glass slides for both experiments, then fixed. After indirect immunoassay (peroxidase antiperoxidase), cells were stained with Harris' haematoxylin.

*Oestrogen receptors* Cells were fixed with 3.7% formaldehyde in PBS, then with pure methanol and acetone. The specific monoclonal antibody used was H222SP gamma from the ER-ICA kit (Abbott).

*Epithelial nature of the cells* Cells were fixed with acetone. An antikeratin monoclonal antibody (KL1-Immunotech) diluted to 1:50 and an anti-epithelial membrane antigen (DAKO, EMA) diluted to 1:25 were used. The same experiments were performed on paraffin sections of the primary tumour of this patient and on a section of a tumour induced in the nude mouse by CAL51.

### *Clonogenic growth in soft agar*

Cells were cultured as described by Salmon *et al.* (1978). In brief, cells were suspended on 0.3% agar in enriched Connaught Medical Research Laboratories Medium 1066 (Grand Island Biological Co., Grand Island, NY, USA) with 15% horse serum (Flow Laboratories, Puteaux, France) in 35 mm Petri dishes containing an underlayer of 0.5% agarose in culture medium. Cells were then incubated at 37°C in a humidified atmosphere of 5% $CO_2$ and 95% air. Plates were examined with an inverted phase microscope 21 days after plating. Positive controls were obtained in the same conditions using melanoma cell line CAL1, which clones in soft agar (Courdi *et al.*, 1983).

### *Tumorigenicity*

Six-week-old athymic nude mice with the Swiss genetic background (IFFA Credo Laboratories, France) were used. Some $2 \times 10^6$ cells in 0.2 ml Ringer lactate solution were inoculated s.c. into both flanks of three mice.

### Results

### *Morphology by light microscopy*

After culturing, epithelial cells appeared rapidly. Figure 1 shows the CAL51 cells as they appeared under light microscopy. These heterogeneous cells (small, polyhedric cells or larger, more rounded cells) showed anchorage-independent growth. They presented visible nuclear abnormalities. The first passage was performed one month after initiation of culture. Cells were maintained in culture until passage 40, then conserved in liquid nitrogen.

**Figure 1** Photomicrograph of cell line CAL51 ( × 70).

### *Doubling time*

CAL51 cells grow exponentially up until day 8, when they reach a plateau phase. The population doubling time was measured during the period of exponential growth: 45 h at passages 15 and 50.

### *Electron microscopy*

CAL51 cells all have a large nucleus with irregular contours and a large nucleolus. The cytoplasm contains abundant ribosomes (free or organised in ergastoplasmic cisternae),

**Figure 2** Electron micrograph of cell line CAL51: **a**, zonula adhaerens (ZO) ( × 17,000); **b**, microfilaments (MF) ( × 17,000).

mitochondria, rather numerous Golgi bodies and some lysosomes. The cells are often contiguous and have zonula-adhaerens junctions (Figure 2a). Certain cells contain numerous bundles of microfilaments organised around electron-free cavities (Figure 2b). Their apical poles are covered with microvilli suggesting glandular luminae.

### *Chromosomal analysis*

*T, R, Q and C-banding* Cytogenetic analysis of cell line CAL51 revealed normal diploidy; the average number of chromosomes per cell analysed was 46 (Figure 3). The karyotype was normal: no structural abnormality was seen by R-banding. Cytogenetic analysis of xenografted CAL51 cells plates *in vitro* gave the same results. Some metaphases were incomplete, and others carried one or two abnormal chromosomes. Since these anomalies were not recurrent, it is very likely that they were generated from cells with normal karyotype at late passages. The tetraploid metaphases or prometaphases had no additional anomalies. R and T-banding (Dutrillaux & Couturier, 1981), allowing an identification of the polymorphism which may exist on the short arms of the acrocentrics, clearly showed that the parental chromosomes were present for pairs 13, 14, 15, 21 and 22 (Figure 4). Q-bands confirmed this interpretation and exhibited a polymorphism for chromosome 3. Finally, chromosomes 9 could be differentiated by their heterochromatin.

**Figure 3** Chromosome number distribution in a sample of 100 CAL51 cells.

**Figure 4** Conservation of heteromorphism of heterochromatic regions. First row, Q-banding; second row, Giemsa staining of the same chromosomes; third row, R-banding of acrocentric chromosomes; fourth row, Q-banding of chromosomes 3 and R-banding after heat denaturation or Brd U incorporation (right) of chromosomes 9.

*High resolution banding* Apart from the fact that a high proportion of tetraploidy was found in CAL51 cells and in xenografted cells as well, no recurrent abnormalities could be observed in the 35 established karyotypes. After synchronisation and BrdU incorporation, a normal replication pattern was observed (Figure 5). In some prometaphases, a high number of bands per cell could be analysed, and yet, no anomalies could be detected. The only abnormal parameter was that a 18–20 h treatment by BrdU was necessary to obtain a replication banding pattern equivalent to a 7 h treatment in lymphocytes. This indicates a very low cell cycle, specially for the late S and G2-phases.

*Immunochemical studies*

*Oestrogen receptors (ER)* The results are negative. This is not surprising because the patient's primary tumour was ER negative.

*Epithelial nature of the cells* Reactivities with antibodies KL1 and anti-EMA were strongly positive (Figure 6a). The same was true for the section of the original tumour and the section of the tumour induced by CAL51 in a nude mouse (Figure 6b).

*Cloning in soft agar*

CAL51 cells had a cloning efficiency of 5.9%.

*Tumorigenicity*

Ten days after inoculation of CAL51 cells, four tumours appeared at six injection sites. The xenografts were excised after 1.5 months and put back in culture. The cells obtained were identical to those of cell line CAL51. Histological examination of the grafted tumours diagnosed differentiated adenocarcinoma (Figure 7a). A histologic section of this patient's tumour is shown in Figure 7b for comparison.

### Discussion

Characterisation of CAL51 cells revealed that these epithelial cells are unquestionably of mammary origin. They are tumorigenic in nude mice and clone in soft agar. Because their phenotype is stable in culture and their doubling time is suitable for kinetics studies, they are a valuable model system for the study of mammary adenocarcinoma. Their main originality is their normal karyotype. CAL51 consists of a population of diploid cells with normal chromosomes after metaphase and high resolution bandings. Since a proportion of metaphases exhibited non-clonal anomalies, such as chromosome gains or losses, or structural rearrangements, it is likely that a certain chromosomal instability exists. In some cultures, these cells undergo endoreduplication without additional anomalies. At least for acrocentrics and chromo-

**Figure 5** Karyotype of cell line CAL51 obtained from xenografted tumour cells (after 13 *in vitro* passages followed by five passages into nude mice). R-bands were obtained by a 20 h BrdU incorporation.

**Figure 6** Immunoperoxidase staining of CAL51 with antikeratin antibodies: **a**, culture on glass slides ( × 700); **b**, section of xenografted tumour ( × 175).

**Figure 7** **a**, Grafted tumour (differentiated adenocarcinoma) ( × 175). **b**, Primary tumour of the patient (poorly differentiated carcinoma) ( × 175).

somes 1 and 9 where it could be investigated no loss of heterozygosity was detected, even for chromosome 13 for which losses frequently occur (Gerbault-Seureau *et al.*, 1987; Lundberg *et al.*, 1987). The phenotype of these cells and their high tumorigenicity indicate that they are not normal contaminating cells, which could have been present in the pleural effusion and which can be maintained *in vitro*. Cytogenetic analysis of the cells from the tumour induced by CAL51 in the nude mouse confirmed the 'normality' and the stability of the karyotype. This fact is not exceptional, and has already been observed by other authors. In his review of human breast cytogenetics, based principally on unbanded cases, Sandberg (1980) stated that diploid metaphases are occasionally observed. Trent (1985) also cited the work by Wolman (1983), who found karyotypically normal diploid cells in primary mammary adenocarcinomas but not in metastatic effusions.

These authors all concluded that acquisition of an abnormal karyotype is indicative of malignant progression rather than initial events in tumorigenesis. As mentioned by Trent (1985) and Smith *et al.* (1985), it could be questioned whether diploid cells are really representative of the tumoral population. In culture since October 1985, cell line CAL51 has conserved a diploid karyotype with normal chromosomes. They still produce tumours in nude mice, which is an argument to consider that they represent the original cancer.

The genetic alterations responsible for the transformation into cancerous cells should exist in the genome of CAL51 cells, but they are not visible on the chromosomes, even after high resolution banding. Molecular studies might demonstrate these alterations, which may be minimal, at difference with most established cancer cell lines in which, the chromosomal damage is so extensive that the most characteristic aberrations are difficult to single out. CAL51 is an ideal model for investigating essential genetic alterations involved in malignant transformation of mammary gland cells.

The authors wish to thank Nancy Rameau, Claude Menghelli and Bernard Fontaine for their assistance in preparation of this manuscript.

### References

CAILLEAU, R., YOUNG, R., OLIVE, M. & REEVES, W.J. Jr (1974). Breast tumor cell lines from pleural effusions. *J. Natl Cancer Inst.*, **53**, 661.

CERVENKA, J. & KOULISCHER, L. (1973). *Chromosomes in Human Cancer*, Gorlin, R. (ed.) p. 132. Charles Thomas: Springfield, IL.

CHU, M., HAGERTY, M., WEIMANN, M. & 6 others (1985). Differential characteristics of two newly established human breast carcinoma cell lines. *Cancer Res.*, **45**, 1357.

COURDI, A., GIOANNI, J., LALANNE, C.M. & 4 others (1983). Establishment, characterization and response to cytotoxic and radiation treatment of three human melanoma cell lines. *In Vitro*, **19**, 453.

DUTRILLAUX, B. & COUTURIER, J. (1981). *La Pratique de l'Analyse Chromosomique*, p. 7. Masson: Paris.

DUTRILLAUX, B. & LEJEUNE, J. (1971). Sur une nouvelle technique d'analyse du caryotype humain. *CR Acad. Sci. Paris*, **272**, 2638.

ENGEL, L.W., YOUNG, N.A., TRALKA, T.S., LIPPMAN, M.E., O'BRIEN, S.J. & JOYCE, M.J. (1978). Establishment and characterization of three new continuous cell lines derived from human breast carcinomas. *Cancer Res.*, **38**, 3352.

GEBHART, E., BRÜDERLEIN, S., AUGUSTUS, M., SIEBERT, E., FELDNER, J. & SCHMIDT, W. (1986). Cytogenetic studies on human breast carcinomas. *Breast Cancer Res. Treat.*, **8**, 125.

GERBAULT-SEUREAU, M., VIELH, P., ZAFRANI, B., SALMON, R. & DUTRILLAUX, B. (1987). Cytogenic study of twelve human near-diploid breast cancers with chromosomal changes. *Ann. Genet.*, **30**, 138.

ISCN (1985). *Cytogenet. Cell Genet.*, **21**, 8.

LAFARGUES, E. & OZZELLO, L. (1958). Cultivation of human breast carcinoma. *J. Natl Cancer Inst.*, **21**, 1131.

LUNDBERG, C., SKOOG, L., CAVENEE, W.K. & NORDENSKJÖLD, M. (1987). Loss of heterozygosity in human ductal breast tumors indicates a recessive mutation on chromosome 13. *Proc. Natl Acad. Sci. USA*, **84**, 2372.

SALMON, S.E., HAMBURGER, A.W., SOENHLEN, B., DURIE, B.G.M., ALBERTS, D.S. & MOON, T.E. (1978). Quantitation of different sensitivity of human tumor stem cells to anticancer drugs. *N. Engl. J. Med.*, **298**, 1321.

SANDBERG, A.A. (1980). *The Chromosomes in Human Cancer and Leukemia*. Elsevier: New York.

SMITH, H.S, LIOTTA, L.A., HANCOCK, M.C., WOLMAN, S.R. & HACKETT, A.J. (1985). Invasiveness and ploidy of human mammary carcinomas in short-term culture. *Proc. Natl Acad. Sci. USA*, **82**, 1805.

SOULE, H.D., VAZQUEZ, J., LONG, A., ALBERTS, S. & BRENNAN, M. (1973). A human cell line from a pleural effusion derived from a breast carcinoma. *J. Natl Cancer Inst.*, **51**, 1409.

TRENT, J.M. (1985). Cytogenetic and molecular biologic alterations in human breast cancer: a review. *Breast Cancer Res. Treat.*, **5**, 221.

VANDEWALLE, B., COLLYN D'HOOGHE, M., SAVARY, J.B. & 5 others (1987). Establishment and characterization of a new cell line (VHB-1) derived from a primary breast carcinoma. *J. Cancer Res. Clin. Oncol.*, **113**, 550.

VIEGAS-PEQUIGNOT, B. & DUTRILLAUX, B. (1978). Une méthode simple pour obtenir des prophases et des prometaphases. *Ann. Génet.*, **21**, 122.

WHITEHEAD, R.H., BERTONCELLO, I., WEBBER, L.M. & PEDERSEN, J.S. (1983). A new human breast carcinoma cell line (PMC42) with stem cell characteristics. I. Morphologic characterization. *J. Natl Cancer Inst.*, **70**, 649.

WOLMAN, S.R. (1983). Karyotypic progression in human tumors. *Cancer Metastasis Rev.*, **2**, 257.

YAMANE, M., NISHIKI, M., KATAOKA, T. & 7 others (1984). Establishment and characterization of a new cell line (YMB-1) derived from human breast carcinoma. *Hiroshima J. Med. Sci.*, **33**, 715.